# Time-Dependent Molecular Changes Following MDMA-Induced Nephrotoxicity

**DOI:** 10.5812/ijpr-145483

**Published:** 2024-05-18

**Authors:** Mehrdad Roghani, Ravieh Golchoobian, Maryam Mohammadian, Farzane Shanehbandpour-Tabari, Zahra Salehi, Saba Gilaki-Bisheh

**Affiliations:** 1 Neurophysiology Research Center, Shahed University, Tehran, Iran; 2 Department of Physiology, Babol University of Medical Sciences, Babol, Iran; 3 Cellular and Molecular Biology Research Center, Health Research Institute, Babol University of Medical Sciences, Babol, Iran; 4 Department of Physiology, School of Medicine, Kermanshah University of Medical Sciences, Kermanshah, Iran; 5 Hematology, Oncology and Stem Cell Transplantation Research Center, Tehran University of Medical Sciences, Tehran, Iran; 6 Research Institute for Oncology, Hematology and Cell Therapy, Tehran University of Medical Sciences, Tehran, Iran; 7 Department of Pathology, School of Medicine, Tehran University of Medical Sciences, Tehran, Iran

**Keywords:** 3,4-Methylenedioxymethamphetamine, Ecstasy, Acute Kidney Injury, Apoptosis, Inflammation

## Abstract

The increasing recreational use of ecstasy (MDMA) poses significant risks to human health, including reports of fatal renal failure due to its adverse renal effects. While MDMA-induced renal toxicity might result from systemic effects, there is also substantial evidence of direct harm to renal tissues by MDMA or its metabolites. The precise mechanisms underlying renal toxicity remain unclear. This study explored the impact of a single intraperitoneal dose of MDMA (20 mg/kg) on rat kidneys. Serum BUN and creatinine levels were evaluated to assess renal function, while TNF-α and TGF-β protein concentrations were measured using ELISA. mRNA levels of Bax, Bcl-xl, and Bcl-2 were quantified using quantitative RT-PCR. Additionally, apoptosis and histopathological changes in renal tissue were examined. Results showed a transient increase in serum BUN and creatinine in MDMA-treated rats. There were decreases in TNF-α and TGF-β levels in the renal tissue. Both pro-apoptotic Bax and anti-apoptotic Bcl-xl gene expressions were significantly reduced, whereas Bcl-2 expression and apoptosis did not show significant changes. No structural alterations were observed in the renal tissues. Overall, this study suggests that the renal adverse effects of MDMA may be mediated through the disruption of cytokine pathways, with notable reductions in TGF-β possibly linked to decreased TNF-α levels.

## 1. Background

The recreational drug ecstasy (3,4-methylenedioxymethamphetamine; MDMA) is frequently misused by youths to enhance sexual excitement, intimacy, and energy levels for prolonged dancing sessions. MDMA's psychological effects are akin to those of the stimulant amphetamine family and the hallucinogenic mescaline (1). The increasing use of MDMA in recent decades, driven by its perceived low toxicity, easy availability, and affordability, has led to a rise in reports of multisystem toxicities and fatal complications (1, 2). There is evidence of renal-related adverse effects, such as hyponatremia and acute kidney injury (AKI), linked to MDMA consumption. AKI is often a complication of acute health issues including malignant hypertension (3), hyperthermia, multi-organ failure, rhabdomyolysis, and disseminated intravascular coagulation (DIC) (4-8). Additionally, post-mortem findings from a case of chronic renal failure following oral ecstasy ingestion revealed necrotizing vasculitis in a renal biopsy, suggesting a direct nephrotoxic effect of MDMA (9). There is also a case report where MDMA ingestion led to transient proximal tubular injury and hyponatremia (10), and studies on primary cultures of rat and human renal proximal tubular cells have shown the direct cytotoxic effects of MDMA or its metabolites on renal injury development (10). Although the link between ecstasy use and acute renal failure has been documented in rodent models (11) and several case reports, the underlying mechanisms remain poorly understood (12). 

## 2. Objectives

This study aimed to explore the potential molecular mechanisms behind MDMA-induced AKI. To this end, we measured TNF-α and TGF-β protein levels and the apoptosis rate in renal tissue of MDMA-exposed rats. Additionally, we assessed the expression rates of the Bcl-2 protein family involved in apoptotic cell death, including mRNA levels of Bax, Bcl-xl, and Bcl-2 in renal tissues.

## 3. Methods

### 3.1. Chemicals and Reagents

MDMA (purity: 99.8%) was sourced from the organic chemistry laboratory at the Faculty of Pharmacy, Tehran University of Medical Sciences, Tehran, Iran. RiboEx and total RNA kits were obtained from GeneAll (Seoul, Korea, catalog no. RR820L). The cDNA synthesis kit and SYBR Premix Ex Taq™ came from Takara Bio Inc (Otsu, Shiga, Japan, catalog no. RR820L). The cell-death detection ELISA kit was procured from Roche (Mannheim, Germany, catalog no. 11544675001). Rat TNF-α ELISA kit and protease inhibitor came from Sigma-Aldrich (St. Louis, MO, USA, catalog no. RAB0480), and the TGF-β1 ELISA kit was from eBioscience (Santa Clara, CA, catalog no. 88–8350). Primers for RT-PCR analysis were synthesized by Bioneer, Korea.

### 3.2. Animals 

Adult male Wistar rats weighing 200 - 250 g were procured from the animal house of Babol University of Medical Science (Babol, Iran). The animals were housed under controlled conditions: Temperature at 24 ± 2°C, a 12/12-h light/dark cycle, and 50 ± 5% humidity, with free access to food and water. All laboratory procedures were approved by the University Ethics Committee (No: IR.MUBABOL.REC.1400.054) and conducted in accordance with the Guidelines for the Care and Use of Laboratory Animals.

### 3.3. Experimental Design 

The rats were randomly divided into two equal groups (n = 12/group). They received either a single dose of MDMA (20 mg/kg, i.p.) or physiological saline (NaCl 0.9%) as the vehicle. The dosage and route of administration were chosen based on previous rodent studies (13-15). The rats were euthanized under deep anesthesia with an intraperitoneal injection of ketamine and xylazine at 6 or 24 hours after treatment to assess study parameters. This timing was selected because the pharmacological effects of MDMA last 4 - 6 hours (16, 17), and previous in vivo and in vitro studies have reported that MDMA induces apoptosis 24 hours following exposure (18-21).

### 3.4. Measurement of Kidney Function 

Blood samples were collected from animals under deep anesthesia. Serum was separated by centrifugation at 3000 rpm for 10 minutes and kidney function markers, including serum BUN and creatinine, were measured using a Sapphire 800 auto analyzer (Cork, Ireland).

### 3.5. Reverse Transcription Polymerase Chain Reaction (RT-PCR) 

Renal tissues were snap-frozen in liquid nitrogen immediately after isolation and stored at -70°C. Total RNA was extracted using RiboEx Total RNA, following the manufacturer’s instructions. The quality and quantity of the extracted RNA were assessed with a NanoDrop 2000 spectrophotometer (Thermo Scientific, Wilmington, DE, USA) at 260 nm and 280 nm wavelengths. RNA samples were treated with DNase I RNase-free solution to eliminate genomic DNA contamination before cDNA synthesis. Complementary cDNA was synthesized from 0.5 µg of total RNA in a 10 µL reaction using a cDNA synthesis kit (Thermo Fisher Scientific), according to the manufacturer’s protocol.

### 3.6. Quantitative PCR

q-Quantitative PCR (q-PCR) was conducted on a StepOnePlus Real-Time PCR System (Applied Biosystems, Carlsbad, CA, USA) using the following conditions: An initial denaturation at 95°C for 30 seconds, followed by 40 cycles of 95°C for 5 seconds and 60°C for 34 seconds. The reaction mixtures, with a total volume of 20 µL, contained 10 µL of SYBR Premix Ex Taq II, 2 µL of cDNA, 0.4 µL each of 10 mM forward and reverse primers, 0.4 µL of ROX Reference Dye, and 6.8 µL of sterile distilled water. GAPDH served as the housekeeping gene for normalizing gene expressions. The relative expression differences between the MDMA-treated and control groups were calculated using the 2^−ΔΔCt^ method. Primers for q-PCR were designed using Primer 3 and verified with BLAST (NCBI). All primer sequences are listed in Table 1. 

**Table 1.** The Primer Sequences Used in the Quantitative Reverse Transcription Polymerase Chain Reaction (qRT-PCR)

mRNA	Primers	Accession Number	Product Size, bp
**GAPDH**		NM_017008.4	121
Forward	5′-TCTCTGCTCCTCCCTGTTCTA-3′		
Reverse	5′-GGTAACCAGGCGTCCGATAC-3′		
**Bax**		NM_017059.2	141
Forward	5′-CTCAAGGCCCTGTGCACTAAA-3′		
Reverse	5′-GGGGGTCCCGAAGTAGGAA-3′		
**Bcl-2**		NM_016993.2	99
Forward	5′-CATCGCTCTGTGGATGACTGA-3′		
Reverse	5′-CTGGGGCCATATAGTTCCACAA-3′		
**Bcl-xl**		NM_001033670.1	109
Forward	5′-GCAGTCAGCCAGAACCCTATC-3′		
Reverse	5′-GGGCTCAACCAGTCCATTGT-3′		

### 3.7. Preparation of Tissue Lysates 

Fresh renal tissue was dissected, homogenized in cold 10% RIPA lysis buffer containing 0.1% Triton X100, 0.1% sodium deoxycholate, 0.1% sodium dodecyl sulfate, and a protease inhibitor cocktail (AEBSF, aprotinin, bestatin, E-64, leupeptin, and EDTA) using an IKA homogenizer (Germany). The homogenates were then centrifuged at 10,000 rpm for 5 minutes at 4°C. The supernatants were stored at -70°C until needed for ELISA assays.

### 3.8. Assessment of Renal TNF-α and TGF-β Levels and Apoptosis 

TNF-α and TGF-β levels, as well as cytoplasmic histone-associated DNA fragments in tissue lysates, were quantified following the manufacturers' instructions for each assay kit. TNF-α and TGF-β were expressed in picograms per milliliter, and DNA fragmentation data were presented in optical density (OD).

### 3.9. Renal Histology 

Six and twenty-four hours after MDMA administration, fresh renal samples were fixed in 10% formalin, processed, and embedded in paraffin. Sections of 5 µm thickness were cut using a rotary microtome and stained with Hematoxylin and Eosin. Pathological evaluation was performed using light microscopy by an observer blinded to the sample identities.

### 3.10. Statistical Analysis 

Data are presented as means ± SEM. An independent *t*-test was used to compare means after verifying data normality with the Shapiro-Wilk test. A P-value of less than 0.05 was considered statistically significant.

## 4. Results

### 4.1. Renal Function 

**Figure 1.**
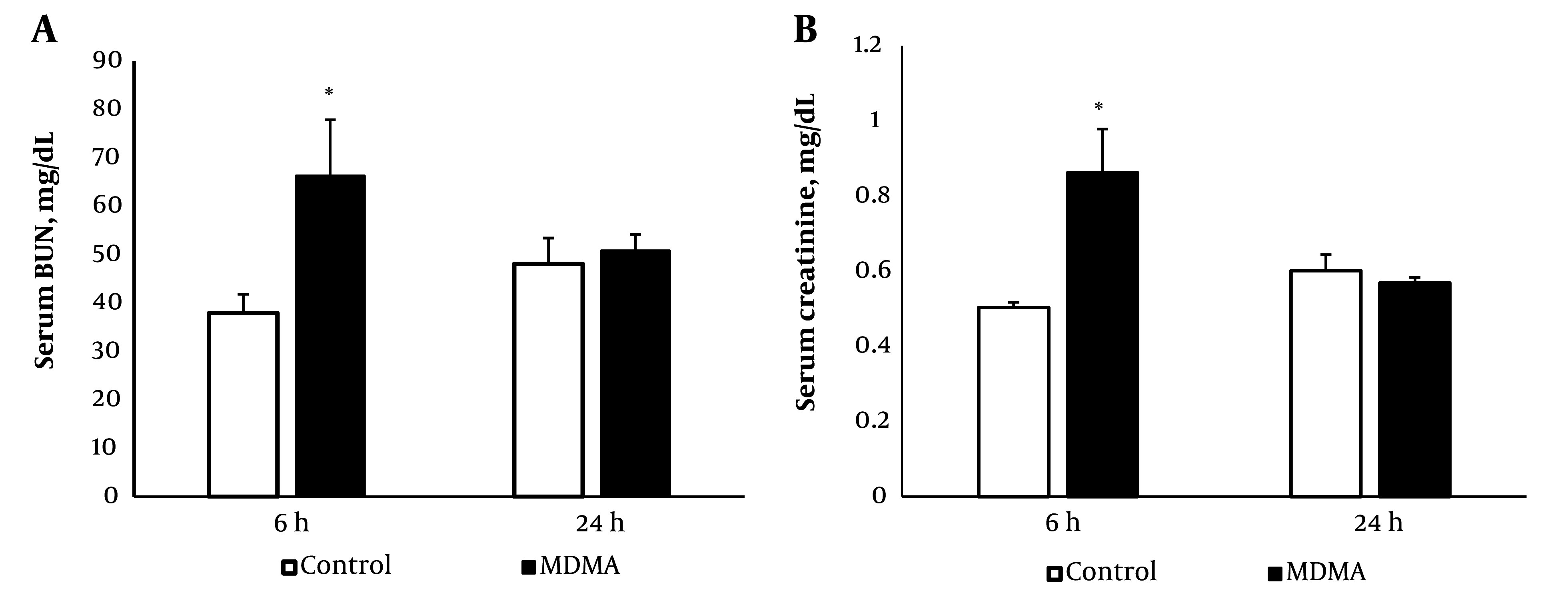
Effect of MDMA (ecstasy) exposure (20 mg/kg, i.p.) on serum BUN (A); and creatinine (B); 6 h and 24 h after injection. Values are expressed as mean ± SEM (n = 5) (* P < 0.05 vs. the control group).

As shown in Figure 1, MDMA administration significantly increased serum BUN and creatinine levels compared to the control group (P < 0.05), with levels returning to baseline within 24 hours.

### 4.2. Reduction of TNF-α and TGF-β Proteins

**Figure 2.**
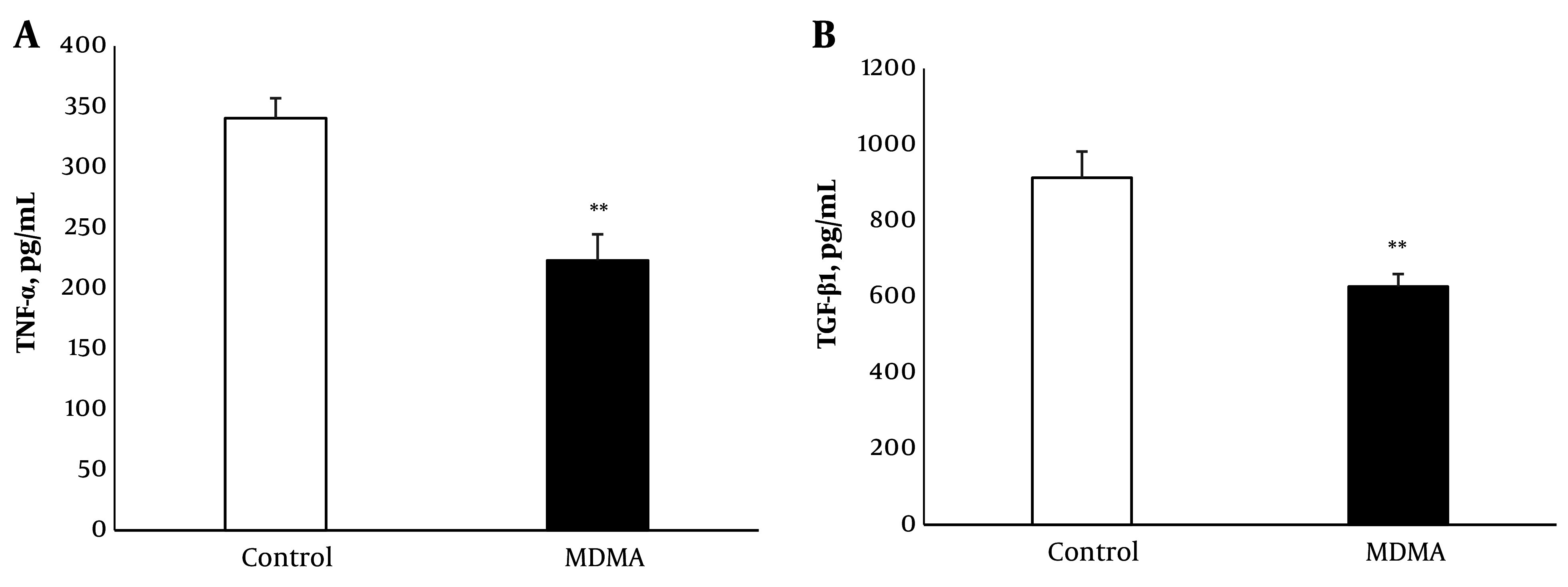
Effect of MDMA (ecstasy) exposure (20 mg/kg, i.p.) on TNF-α (A); and TGF b1 (B); in kidney tissue. Values are expressed as mean ± SEM (n = 5 - 6) (** P < 0.01 vs. the control group).

Renal TNF-α levels decreased significantly (P < 0.01) 6 hours after MDMA injection compared to controls. Similarly, renal TGF-β protein levels were significantly reduced (P < 0.01) in response to MDMA (Figure 2A and B).

### 4.3. Bcl-xl, Bax, and Bcl-2 Gene Expression

**Figure 3.**
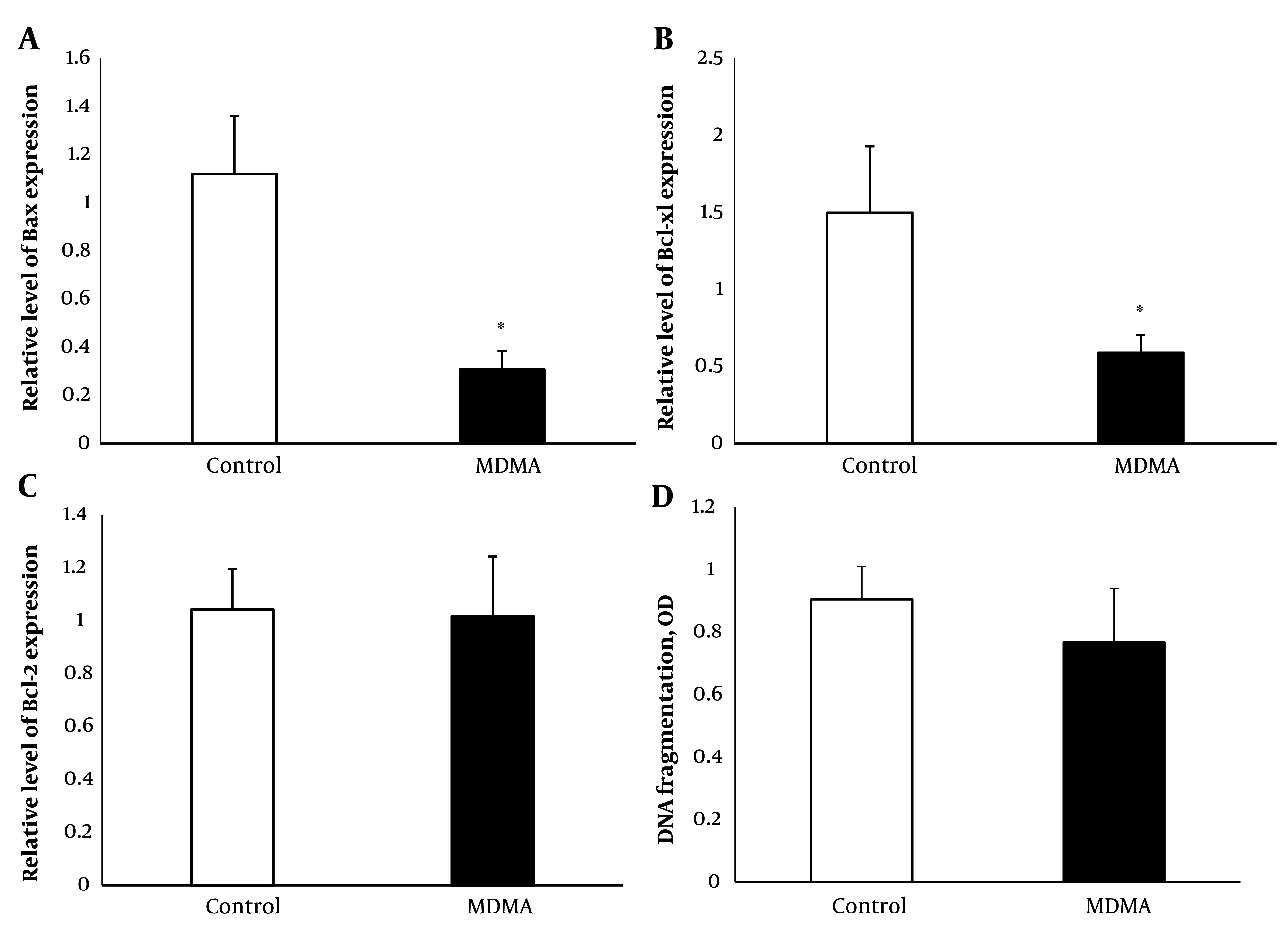
Effect of MDMA (ecstasy) exposure (20 mg/kg, i.p.) on Bax (A); Bcl xl (B); and Bcl 2 (C); relative expression and DNA fragmentation (D). Values are expressed as mean ± SEM (n = 5) (* P < 0.05 vs. the control group).

A significant reduction in the expression of Bax and Bcl-xl was observed (P < 0.05) in renal samples 24 hours after MDMA administration compared with the control group (Figures 3 and 2B). Additionally, real-time PCR results indicated that there were no significant changes in Bcl-2 expression in the MDMA group compared to the control group (Figure 3C). 

### 4.4. Renal Apoptosis

The impact of MDMA on renal tissue apoptosis was assessed using a cytoplasmic histone-associated DNA fragments assay, recognized as a valid indicator of apoptosis (22), 24 hours post-MDMA injection. There was no significant difference in renal tissue apoptosis between MDMA-treated animals and the control group (Figure 3D). 

### 4.5. Histological Results 

**Figure 4.**
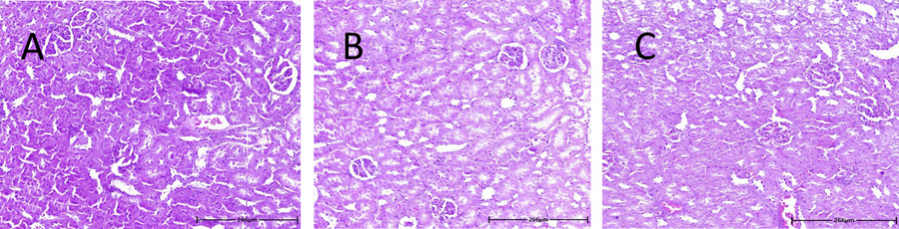
Hematoxylin and eosin (H&E)-stained kidney sections from rat treated with vehicle (A); or MDMA (20 mg/kg, i.p.) after 6 (B); and 24 (C); hours.

Hematoxylin and Eosin staining was utilized to examine the effects of MDMA on kidney tissue. The results showed no noticeable histological changes in rat renal tissue 6 and 24 hours after MDMA administration compared to the control group (Figure 4). 

## 5. Discussion

The results of this study demonstrated a decrease in renal function following MDMA administration, evidenced by a reversible increase in serum BUN and creatinine levels. A reduction in TNF-α, TGF-β, Bax, and Bcl-xl levels was observed in kidney tissue after MDMA injection. BUN and creatinine levels were found to increase 4 hours post-MDMA treatment, with creatinine returning to baseline within 24 hours (23), aligning with our findings of MDMA-induced transient renal dysfunction. Kwon et al. reported a case of acute transient proximal tubular injury related to MDMA consumption, characterized by polyuria, glycosuria, and solute diuresis with low tubular reabsorption of phosphorus, persisting for up to three days. Although this indicates direct cytotoxic effects of MDMA or its metabolites on renal tubules, the mechanisms underlying ecstasy-induced damage remain unclear (10). Further, studies on the direct toxic effects of MDMA and its metabolite methylenedioxyamphetamine (MDA) on primary cultures of renal proximal tubular cells did not reveal any significant decline in cell viability. However, some of MDMA's putative metabolites have been shown to increase cell death in renal proximal tubular cells (24). This inconsistency in the direct cytotoxic effects of MDMA necessitates further experimental research.

TNF-α, an inflammatory and multifunctional cytokine, is produced in the kidney by podocytes, mesangial cells, and renal tubular epithelial cells (25). Some studies have demonstrated the immunosuppressive effects of MDMA (26, 27). The significant decrease in renal TNF-α levels 6 hours post-drug injection aligns with the immunosuppressive effects of MDMA, similar to the effects observed in a single dose study like ours, which showed a sustained immunosuppressive effect lasting at least 6 hours following injection (28). Additionally, this decrease mirrors findings from our previous study where liver tissue TNF-α also decreased in MDMA-exposed animals compared to the control group (29). Although numerous reports highlight the role of TNF-α overexpression in the pathogenesis of renal disease (30), the mechanisms by which normal TNF-α levels contribute to preserving kidney function have received scant attention. Notably, administration of exogenous TNF-α in spontaneous models of lupus in B/W mice, which have low endogenous TNF-α production, has shown a protective role for this cytokine in delaying the progression of renal disease (31). Moreover, there are reports that TNF inhibitor therapy can lead to nephrotoxicity, manifesting as glomerulonephritis and AKI (32-34). Supporting this hypothesis, a renal biopsy from a case of fatal renal failure secondary to MDMA use revealed significant changes in small arterioles and arteries with few inflammatory cells (9). The pathogenic role of anti-TNF-α treatment is underscored by the close temporal relationship between the onset of renal complications and drug use, and by the improvement in laboratory abnormalities and clinical symptoms after drug discontinuation (35).

The results of this study showed a significant reduction in renal TGF-β1 levels 24 hours post-MDMA injection. However, a previous clinical study reported an increase in serum TGF-β1 levels following drug administration (36), highlighting a possible discrepancy related to different sample types. It should also be noted that TGF-β1 levels in other peripheral tissues such as the liver and lungs did not change in our experimental model (data not shown). The reduction in renal TGF-β may be partly due to the TNF-α suppressive effect of MDMA. This is supported by observations that TNF-α-neutralized rats exhibit a marked decrease in renal TGF-β production (37, 38). Correspondingly, Kassiri et al. reported that TNF-α and TGF-β reciprocally affect each other's expression. Their study showed that blocking either of these cytokines in mice significantly reduces the induction of the other (39). Several studies have indicated that the overexpression of the fibrogenic cytokine TGF-β contributes to the development of renal disease (40-42). Yet, the renal protective and functional actions of TGF-β have also been reported (43). Since nephrons, glomeruli, and renal arterioles express TGF-β, this cytokine plays a crucial role in maintaining the structural and functional homeostasis of the kidneys (44). The protective role of TGF-β was demonstrated by Guan et al. in a mouse model of renal ischemia-reperfusion injury, where the knockout of TGF-β1 aggravated kidney injury (45). Additionally, it has been shown that TGF-β protects tubular epithelial cells against H_2_O_2_-induced necrosis (46).

Apoptosis significantly contributes to various renal diseases, particularly in cases of drug-induced nephrotoxicity. Drug-induced renal cell apoptosis predominantly occurs through the intrinsic pathway, which is regulated in part by the pro- and anti-apoptotic members of the Bcl-2 family (47). The anti-apoptotic members, Bcl-2 and Bcl-xL, preserve mitochondrial outer membrane integrity by binding to the pro-apoptotic protein Bax, which prevents mitochondrial cytochrome c release and maintains mitochondrial membrane integrity. Previous studies have demonstrated the role of reduced anti-apoptotic Bcl-xL in MDMA-induced apoptosis in hepatocyte and hepatic stellate cell lines, with no change in Bax protein levels (18), and in rat neocortical neuronal cell lines without altering mRNA levels of Bax and Bcl-2 (48). This study found that MDMA reduces the gene expression of both Bcl-xL and Bax in renal tissue. These findings align with evidence suggesting that while Bcl-xl and Bcl-2 proteins both serve anti-apoptotic functions, their protein expression is regulated by independent mechanisms, leading to dissociation between their expression changes (49-51). The under-expression of the pro-apoptotic Bax may protect the kidney from MDMA-induced apoptosis, despite a reduction in Bcl-xL mRNA. This concept is supported by Wei et al., who used a proximal tubules Bax knockout model and found that Bax deficiency protected mice from ischemic acute kidney injury. In their model, tubular apoptosis was blocked during ischemic AKI, although tubular necrosis remained unaffected (52).

Although it has been previously suggested that events outside the kidney contribute to the renal adverse effects of ecstasy, this study shows that ecstasy induces molecular changes in kidney tissue that could potentially predispose the kidney to malfunction. Alternatively, these molecular alterations could be part of the kidney's compensatory mechanisms against MDMA-induced adverse effects, though further verification of this hypothesis is needed. Supporting the notion of MDMA's direct nephrotoxicity, Hurault de Ligny et al. (53) reported the early loss of two renal grafts from the same donor—a 21-year-old woman who was a regular ecstasy user for two years. Since immunological complications were the primary cause of early graft rejection, pre-existing vascular lesions in the grafts due to MDMA consumption were proposed as the possible cause of necrotizing vasculitis that led to the loss of both grafts during the first post-transplant week in the absence of any inflammatory elements.

The results of this study demonstrate that kidney tissue TNF-α levels decrease due to the immunosuppressive effects of MDMA. This reduction in TNF-α was associated with decreased TGF-β protein expression, which may partly contribute to the nephrotoxic effects of MDMA. Furthermore, MDMA might exert its effects through a reduction in Bax mRNA. MDMA causes reversible renal dysfunction without structural damage, which was associated with lower TNF-α expression. This immunosuppressive effect may be partly due to reduced renal TGF-β production below normal levels, resulting in altered kidney homeostasis. However, the attenuation of Bcl-xl expression as an anti-apoptotic regulator in the induction of MDMA-induced apoptosis is likely prevented by the downregulation of Bax expression. Further studies are needed to assess MDMA-induced renal adverse effects to determine whether these molecular changes are due to its direct effect on the kidney or its effects secondary to immune dysregulation.

## Data Availability

All the data obtained and/or analyzed during the current study were available from the corresponding authors on reasonable request.
